# Concurrent Occurrence of Port-Wine Stain and Glaucoma in Sturge-Weber Syndrome: A Case Report

**DOI:** 10.7759/cureus.37451

**Published:** 2023-04-11

**Authors:** Karthik Rajaram Mohan, Saramma Mathew Fenn, Ravikumar Pethagounder Thangavelu

**Affiliations:** 1 Oral Medicine and Radiology, Vinayaka Mission's Sankarachariyar Dental College, Vinayaka Mission's Research Foundation (Deemed to be University), Salem, IND

**Keywords:** glaucoma, trigeminal nerve, laser-therapy, chatgpt, port-wine stain

## Abstract

Sturge-Weber syndrome (SWS) is a rare neurological disorder that is present at birth. It is characterized by a reddish-purple birthmark on the face, typically on one side of the forehead and upper eyelid, and sometimes involving the scalp and ear. This birthmark, called a port-wine stain, is caused by an abnormal buildup of blood vessels in the skin. SWS can also cause neurological problems such as seizures, developmental delays, and problems with vision and coordination. Treatment for SWS typically includes a combination of medications to control seizures and other symptoms, as well as laser therapy or surgery to reduce the appearance of the birthmark. Additionally, physical therapy and other therapies can help improve vision and coordination. It is important to note that the symptoms and severity of SWS can vary widely from person to person, and early diagnosis and treatment can help improve outcomes.

## Introduction

A port-wine stain (PWS) is a type of birthmark that appears as a reddish or purple discoloration of the skin [1]. It is caused by an abnormal buildup of blood vessels in the affected area and is typically present at birth [1]. PWS can occur anywhere on the body, but most commonly appears on the face, head, and neck [1]. PWS is a benign (noncancerous) condition and does not cause any physical discomfort [1]. However, it can be cosmetically disfiguring and can lead to psychological and social problems for the affected individual [1]. Sturge-Weber syndrome (SWS) is a rare, nonhereditary developmental phakomatoses characterized by hamartomatous vascular proliferation nevus-flammeus (port-wine stain) involving the face, limbs and trunk along the distribution of branches of trigeminal nerve, glaucoma or vascular angiomas in the eye [1]. Aim of study: It is essential for ophthalmic evaluation in patients clinically presenting with port-wine stain on the face to screen for glaucoma or vascular angiomas affecting the eye, which if neglected can lead to blindness.

## Case presentation

**Figure 1:**
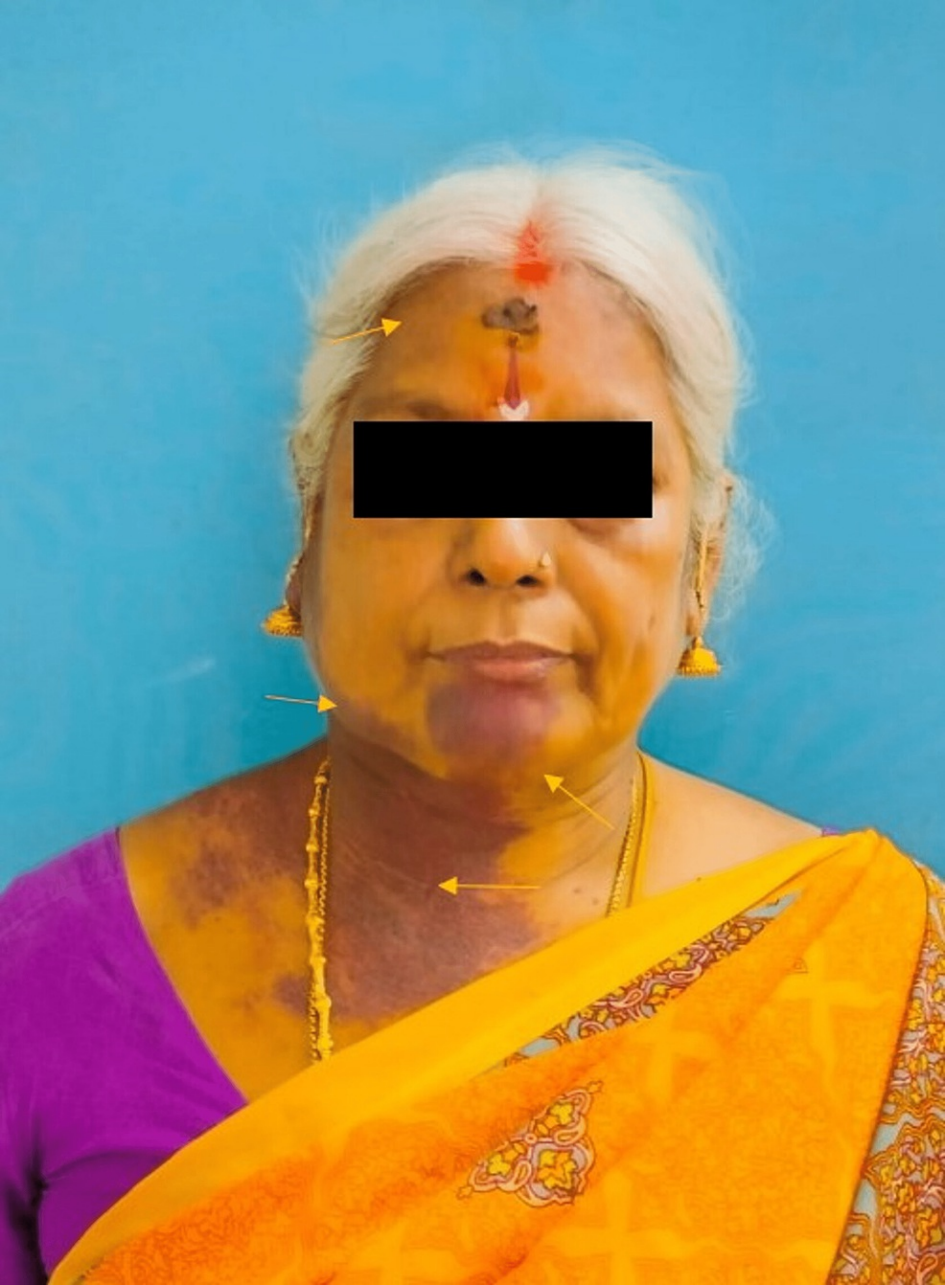
Extraoral examination revealed right-side facial asymmetry and purplish discolourations (port-wine stains) present only on the right side of the face, forehead and neck.

A 47-year-old female came to our department for a routine dental checkup. Since birth, she had multiple discrete purple discolorations on the right side of her chin, neck, and lower limb. She gave a history of blurred vision for the past one year. On general examination, the patient's vital signs were stable. On extraoral examination, facial asymmetry was present on the right side of her face due to soft tissue hypertrophy, along with numerous discrete areas of purple lesions covering the right side of the patient's frontal region of the face, the eyelid, around the nose, chin, and the lips (Figure 1).

**Figure 2:**
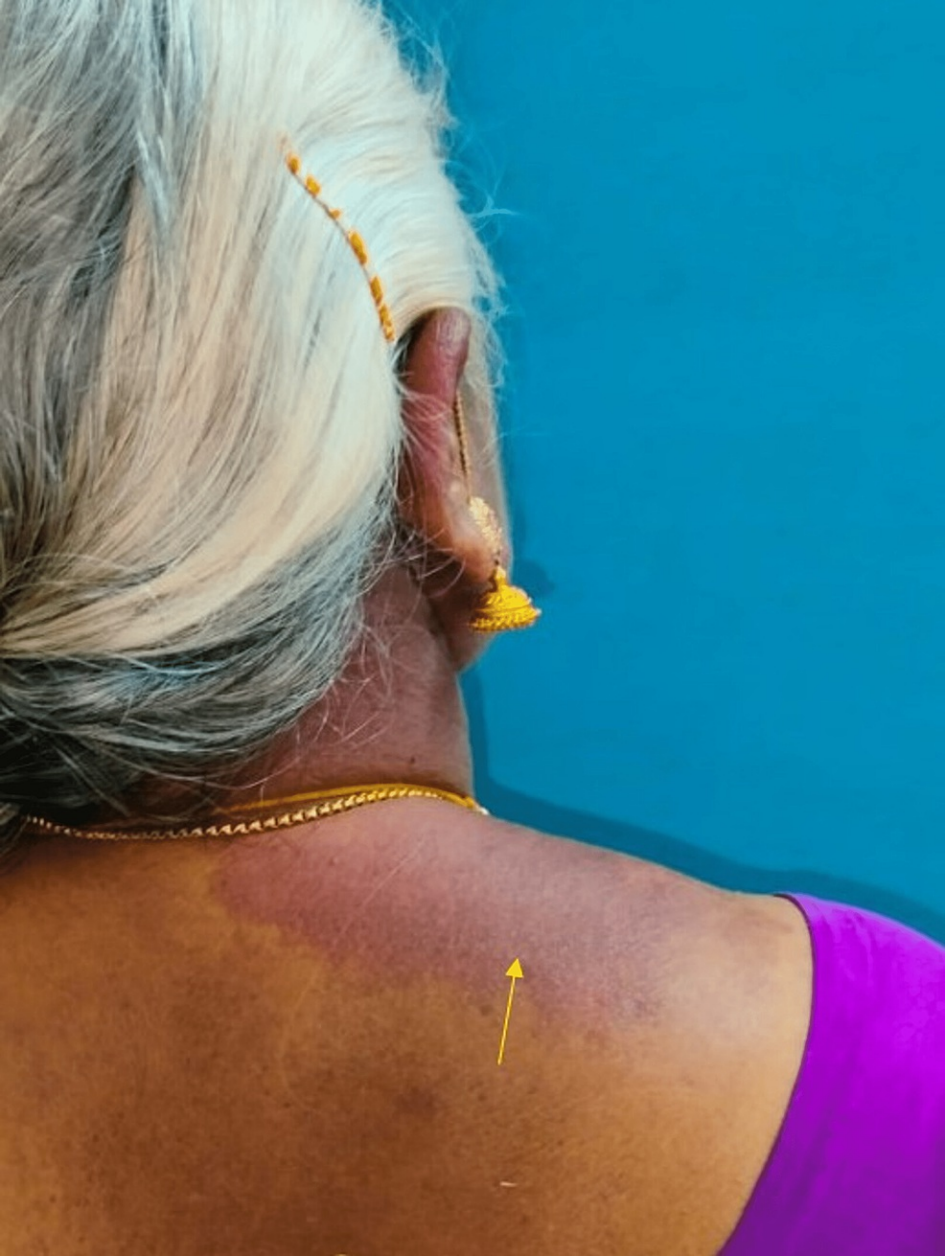
Purplish areas representing port-wine stains on right side of back of neck and shoulder.

These purplish lesions were evocative of port-wine stains (nevus flammeus) involving the back of the neck and shoulder that did not cross the midline (Figure 2). Nevus flammeus or port-wine stain is a benign congenital capillary malformation usually presenting as a unilateral pink, purplish or bright red patch anywhere on the body [2].

**Figure 3:**
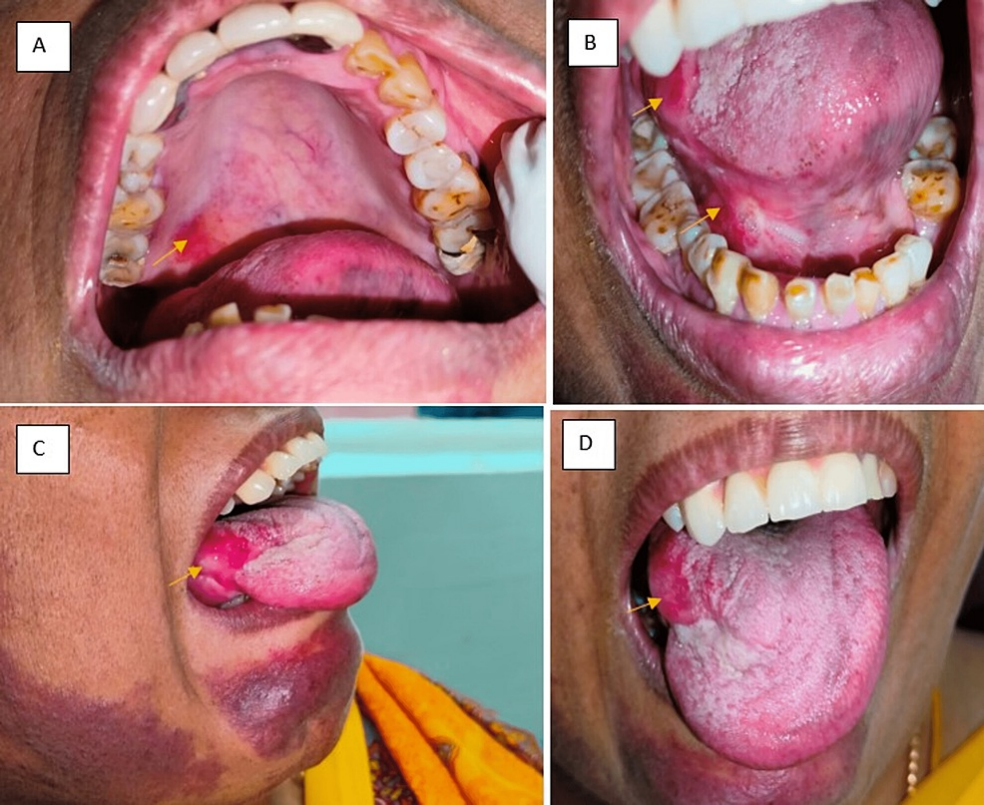
Bright red areas representing fine capillary hemangiomas involving the A) right side hard palate, B) tongue C) floor of the mouth D) increased size of right half of tongue.

Intraoral examination revealed asymptomatic bright-red flat areas of discoloration present from birth involving the right postero-lateral border on the hard palate extending laterally 1.5 cm away from the palatal aspect of free or marginal gingiva in relation to 17 tooth region, medially 2 cm away from mid-palatine raphe region, posteriorly extend till the right posterolateral junction of the hard and soft palate and right ventral surface of the floor of the mouth in relation to the lingual aspect of 44,45,46 tooth region, bright red and increased size of right lateral border of the tongue (Figure 3).

**Figure 4:**
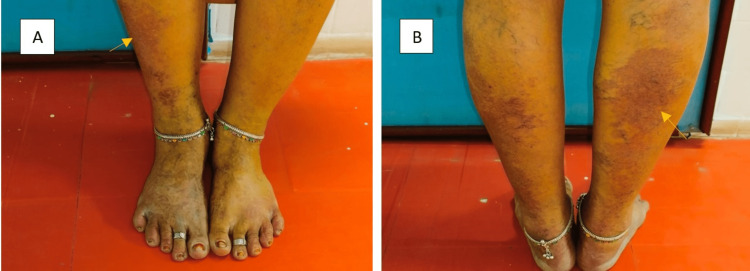
Purplish port-wine stains on the A) flexor and B) extensor aspects of right lower limb.

Similar purplish lesions were also present on the flexor and extensor aspects of the right lower limb (Figure 4). The skin lesions were characterized by flat, non-elevated, purplish colour representing port-wine stains [3]. The oral lesions were bright red in colour due to the presence of oxygenated blood in fine hamartomatous capillaries [3]. The differential diagnoses of Sturge-Weber syndrome include tuberous sclerosis, Klippel-Trenaunay syndrome, Von Hippel-Lindau (VHL) syndrome, Wyburn-Mason syndrome, neurofibromatosis, PHACE syndrome, Cobb syndrome, Maffucci syndrome, Gorham-Stout syndrome, and Parkes Weber syndrome [3]. Hypomelanotic macules (greater than or equal to three, at least 5 mm in diameter), angiofibroma (greater than or equal to three) or fibrous cephalic plaque, ungual fibromas (greater than or equal to two ), and shagreen patch are the four main characteristics of tuberous sclerosis [3]. The three minor characteristics of tuberous sclerosis are intraoral fibromas, "confetti" skin lesions, and dental enamel pits [3]. Klippel-Trenaunay syndrome is characterized by port-wine stains on the limbs, and overgrowth of soft tissue and bones with venous malformations [3]. Hemangioblastomas of the brain, spinal cord, and retina, renal cysts, clear cell renal cell carcinoma, pancreatic cysts, neuroendocrine tumours, endolymphatic sac tumours, and epididymal and wide ligament cysts are the hallmarks of VHL syndrome [3]. Wyburn-Mason syndrome, often referred to as racemose angioma, is a congenital, non-heritable neurocutaneous syndrome or phakomatoses that manifests as numerous arteriovenous malformations and primarily affects the face and brain [3]. PHACE syndrome is characterized by posterior fossa malformations, hemangioma, arterial anomalies, coarctation of the aorta/cardiac defects, and eye abnormalities with port-wine stains involving the face [3]. Cobb syndrome is characterized by the presence of port-wine stains involving the thigh and buttocks with angiomas within spinal canal resulting in paraplegia [3]. Mafucci syndrome is characterized by multiple benign enchondromas affecting bone with hemangiomas of the skin [3]. Gorham-Stout syndrome (vanishing bone disease) is characterized by destruction of osseous matrix due to vascular proliferation in bone resulting in bone pain [3]. Parkes-Weber syndrome is characterized by high-flow arterio-venous malformations of the limb [3].

**Figure 5:**
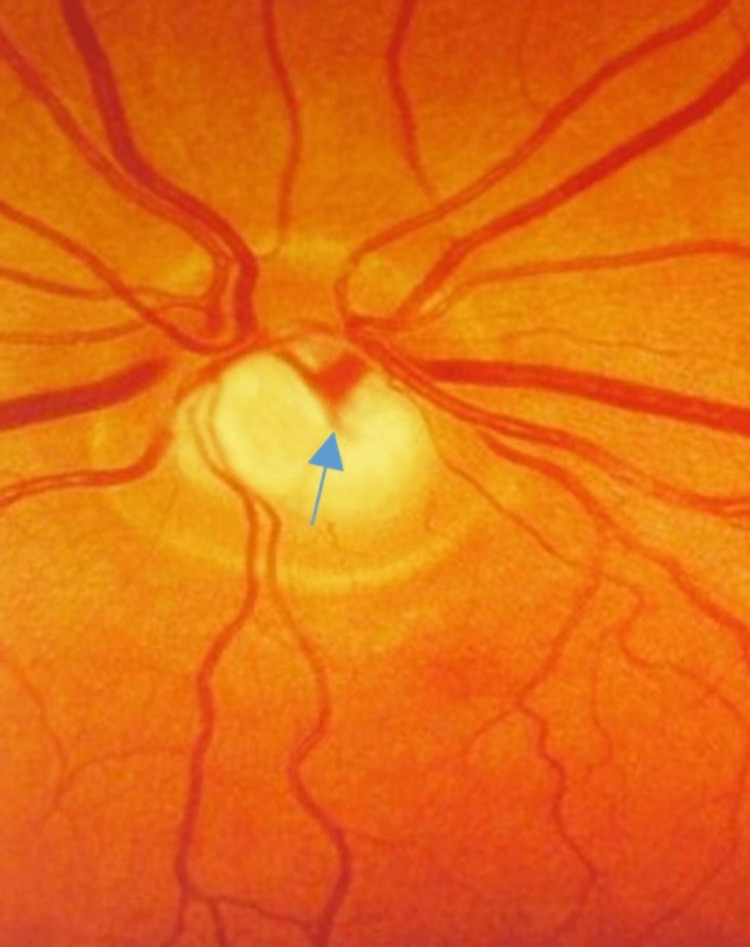
Fundoscopic examination revealed " bayoneting" of retinal vessels on the right eye due to glaucoma.

Fundoscopic examination of the right eye revealed "bean-pot cupping" or "bayoneting" of retinal vessels suggestive of advanced glaucoma (Figure 5).

SWS is diagnosed by the presence of port-wine stains on the skin of the face, forehead, and cheek with a characteristic dermatomal involvement along the distribution of branches of trigeminal nerve and glaucoma. Our patient also had purplish lesions representing port-wine stains along the dermatomal involvement of the trigeminal nerve and on the back of the neck and limbs and glaucoma. The patient was diagnosed with Sturge-Weber syndrome and glaucoma. For the treatment of glaucoma, netarsudil combined with latanoprost (0.02%/0.005%) ophthalmic solution was prescribed at a once-daily dosage for a period of one year [4]. The syndrome, its prognosis, its consequences, and the significance of continuing ophthalmic medication were all explained to the patient. She was counseled to get the port-wine stain removed with pulsed-dye laser photocoagulation.

## Discussion

**Figure 6:**
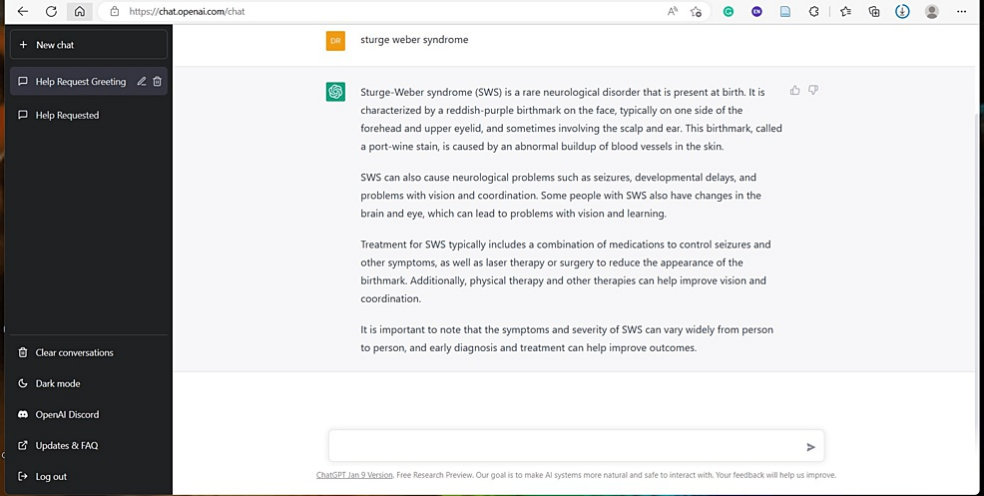
ChatGPT AI tool on Sturge Weber syndrome.

SWS is a rare neurological disorder that is present at birth [1]. It is characterized by a reddish-purple birthmark on the face, typically on one side of the forehead and upper eyelid, and sometimes involving the scalp and ear [1]. This birthmark, called a port-wine stain, is caused by an abnormal buildup of blood vessels in the skin and the eye which can lead to problems with vision (Figure 6) [1].

**Figure 7:**
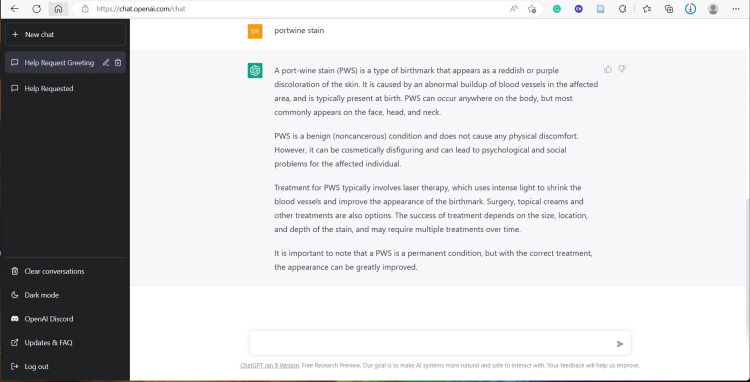
ChatGPT AI tool on port-wine stain

PWS is a birthmark that appears as a reddish or purplish discolouration of the skin [2]. It is caused by an abnormal buildup of blood vessels in the affected area, and is typically present at birth. PWS can occur anywhere in the body, but most commonly appears on the face, head, and trunk [2]. Treatment for PWS typically involves laser therapy, which uses intense light to shrink the blood vessels and improve the appearance of the birthmark [2]. The success of treatment depends on the size, location, and depth of the stain, and may require multiple treatments over time (Figure 7) [2].

Clinical significance

**Figure 8:**
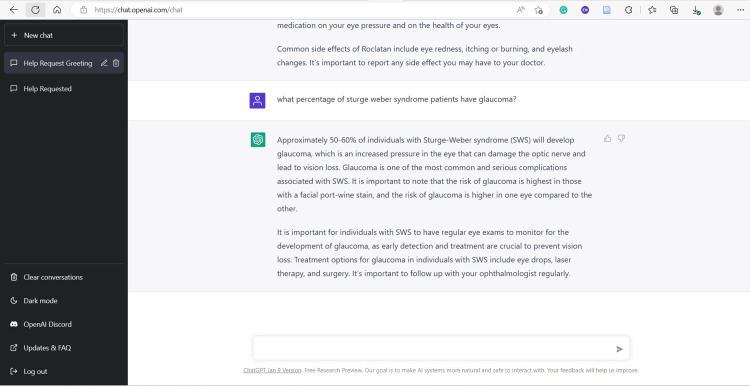
ChatGPT AI tool on Sturge Weber syndrome and glaucoma.

Approximately 50-60% of individuals with SWS will develop glaucoma, which is an increased pressure in the eye that can damage the optic nerve and lead to vision loss [3]. Glaucoma is one of the most common and serious complications associated with SWS [3]. It is important to note that the risk of glaucoma is highest in those with a facial port-wine stain, and the risk of glaucoma is higher in one eye compared to the other [3]. It is important for individuals with SWS to have regular eye exams to monitor for the development of glaucoma, as early detection and treatment are crucial to prevent vision loss (Figure 8) [3]. 

**Figure 9:**
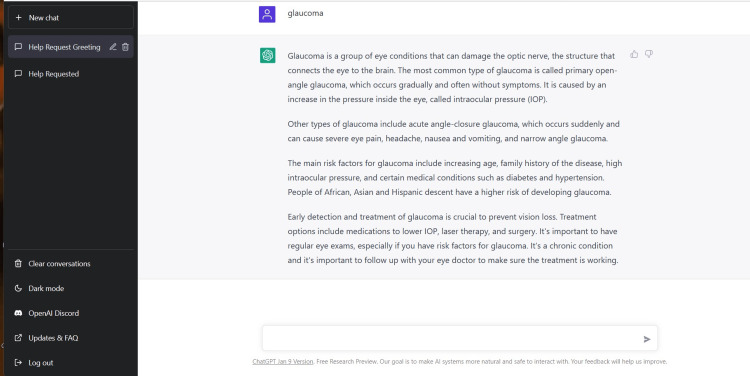
ChatGPT AI tool on glaucoma.

Glaucoma is a group of eye conditions that can damage the optic nerve, the structure that connects the eye to the brain [3]. The most common type of glaucoma is called primary open-angle glaucoma, which occurs gradually and often without symptoms [3]. It is caused by an increase in the pressure inside the eye, called intraocular pressure (IOP). Other types of glaucoma include acute angle-closure glaucoma, which occurs suddenly and can cause severe eye pain, headache, nausea and vomiting, and narrow-angle glaucoma [3]. The main risk factors for glaucoma include increasing age, family history of the disease, high intraocular pressure, and certain medical conditions such as diabetes and hypertension. Early detection and treatment of glaucoma are crucial to prevent vision loss (Figure 9) [3].

**Figure 10:**
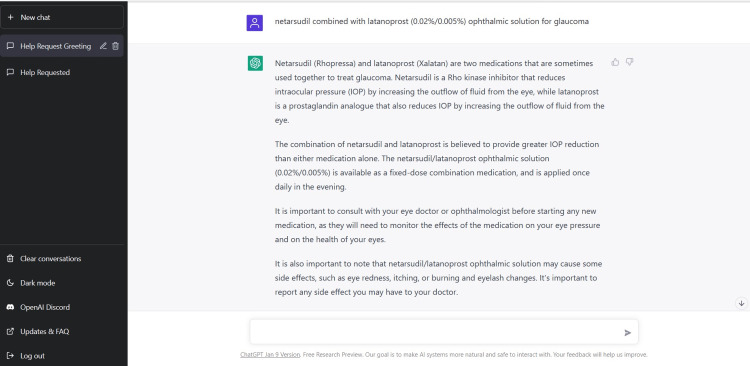
ChatGPT AI tool on netarsudil and lantoprost opthalmic solution to treat glaucoma.

Roclatan (netarsudil/latanoprost ophthalmic solution) is a fixed-dose combination medication prescribed for the treatment of open-angle glaucoma and ocular hypertension [4]. It contains two active ingredients, netarsudil and latanoprost. Netarsudil is a Rho kinase inhibitor that reduces IOP by increasing the outflow of fluid from the eye, while latanoprost is a prostaglandin analogue that also reduces IOP by increasing the outflow of fluid from the eye [4]. The combination of netarsudil and latanoprost is believed to provide greater IOP reduction than either medication alone [4]. Netarsudil/latanoprost ophthalmic solution (0.02%/0.005%) is available as a fixed-dose combination medication, and is applied once daily in the evening (Figure 10).

**Figure 11:**
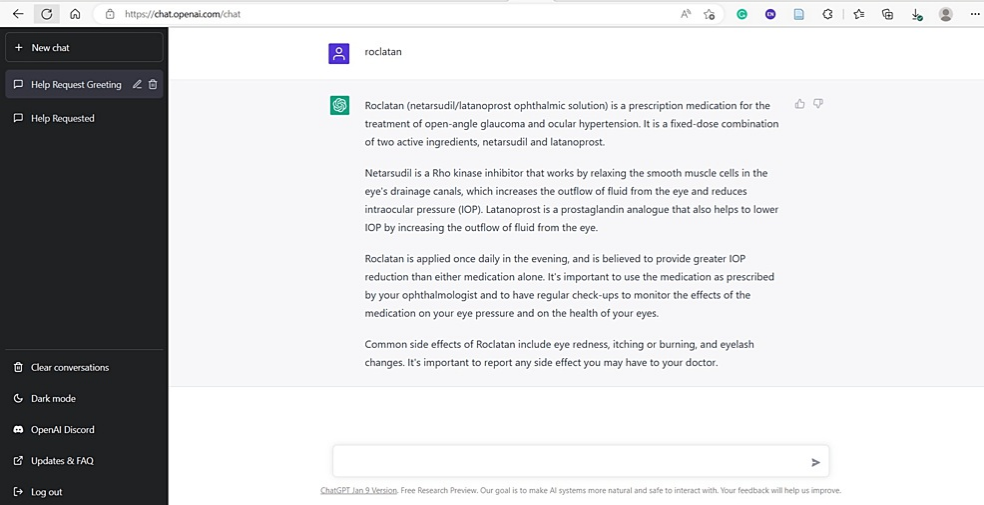
ChatGPT AI tool on drug Roclatan.

The side effects of netarsudil/latanoprost ophthalmic solution may cause some side effects, such as eye redness, itching, or burning sensation (Figure 11) [4]. 

## Conclusions

Dentists must be aware of the clinical signs and symptoms of Sturge-Weber syndrome, such as port-wine stains on the face, neck, tongue, and hard palate. Patients with port-wine stains must have their opthamologic examination to rule out glaucoma or choroidal hemangiomas, which can lead to blindness if unattended on time. By diagnosing glaucoma early in this patient, blindness is prevented, which improves the patient's quality of life.

## Figures and Tables

**Figure 12:**
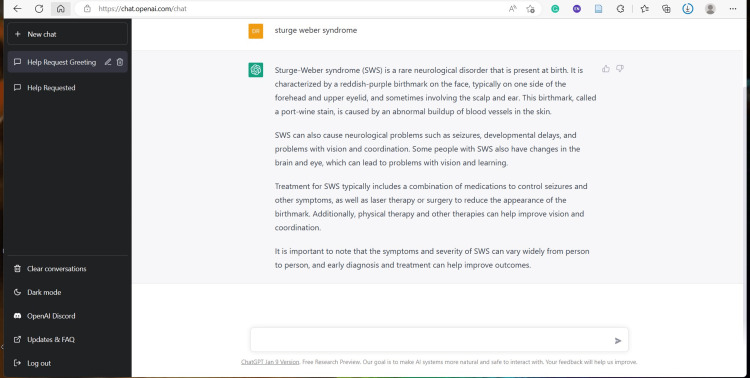
ChatGPT tool on Sturge Weber Syndrome

## Appendices

 I thank ChatGPT AI tool for its assistance in writing this case report (Figure 12).

## References

[1] Raval DM, Rathod VM, Patel AB, Sharma B, Lukhi PD (2022). Sturge-Weber syndrome: a rare case report. Cureus.

[2] Van Trigt WK, Kelly KM, Hughes CC (2022). GNAQ mutations drive port wine birthmark-associated Sturge-Weber syndrome: a review of pathobiology, therapies, and current models. Front Hum Neurosci.

[3] Poliner A, Fernandez Faith E, Blieden L, Kelly KM, Metry D (2022). Port-wine birthmarks: update on diagnosis, risk assessment for Sturge-Weber syndrome, and management. Pediatr Rev.

[4] (2023). Rocklatan (netarsudil/latanoprost). https://www.medicalnewstoday.com/articles/rocklatan.

